# Association of CHADS_2_ and CHA_2_DS_2_-VASc Scores with Left Atrial Thrombus with Nonvalvular Atrial Fibrillation: A Single Center Based Retrospective Study in a Cohort of 2695 Chinese Subjects

**DOI:** 10.1155/2017/6839589

**Published:** 2017-03-08

**Authors:** J. Huang, SL. Wu, YM. Xue, HW. Fei, QW. Lin, SQ. Ren, HT. Liao, XZ. Zhan, XH. Fang, L. Xu

**Affiliations:** ^1^Department of Cardiology, Guangdong Cardiovascular Institute, Guangdong General Hospital, Guangdong Academy of Medical Sciences, Guangzhou, China; ^2^School of Public Health, Sun Yat-sen University, Guangdong, China; ^3^School of Public Health, The University of Hong Kong, Pokfulam, Hong Kong

## Abstract

The main mechanism of the CHADS_2_ and CHA_2_DS_2_-VASc scores to predict stroke in nonvalvular atrial fibrillation (NVAF) is still controversial. We evaluated the association of the CHADS_2_ and CHA_2_DS_2_-VASc scores with left atrial thrombus (LAT) as detected by transesophageal echocardiographic (TEE) and compared the predictive ability of these risk stratification schemes with nonvalvular atrial fibrillation (NVAF). Data from 2,695 consecutive NVAF patients in whom TEE was performed for screening LAT from July 2007 to February 2014 were analyzed. Only 3% of the subjects had LAT. Presence of LAT was not significantly associated with either CHADS_2_  (*P* = 0.07) or CHA_2_DS_2_-VASc score (*P* = 0.12). The area under the curve (AUC) concerning LAT prediction using CHADS_2_ and CHA_2_DS_2_-VASc was 0.574 and 0.569, respectively. A composition model includes previous stroke or transient ischemic attack, nonparoxysmal AF, moderate to severe left ventricular systolic dysfunction, left atrial enlargement, and cardiomyopathy which improved the discrimination significantly (AUC = 0.743). In our cohort, both CHADS_2_ and CHA_2_DS_2_-VASc scores were of limited value for predicting LAT in patients with NVAF. This questions the CHADS_2_/CHA_2_DS_2_-VASc score predicting stroke mainly through the mechanism of cardiogenic embolism. A scoring scheme combining clinical and echocardiographic parameters may better predict LAT as a surrogate for cardioembolic risk in NVAF patients.

## 1. Introduction

CHADS_2_ and CHA_2_DS_2_-VASc score is the most commonly used atrial fibrillation stroke risk stratification schemes. The potential mechanism by which they predict stroke in nonvalvular atrial fibrillation (NVAF) is still controversial. Previous studies have shown that they may predict stroke through the mechanism of cardiogenic embolism because the prevalence of left atrial thrombus (LAT) increased with ascending CHADS_2_ and CHA_2_DS_2_-VASc scores [1, 2]. Current guidelines focused on anticoagulant therapy for stroke prevention in NVAF depend on CHADS_2_ and CHA_2_DS_2_-VASc scores [3–5]. However, many studies showed that they may also predict stroke through the mechanism of atherosclerosis [6–8]. Which of these two mechanisms dominates is still not clear. LAT is thought to be the material basis of cardiogenic embolism in atrial fibrillation. Establishing the relationship between LAT and CHADS_2_/CHA_2_DS_2_-VASc scores may help clarify such issues. Hence, we conducted the current study to examine the association of CHADS_2_/CHA_2_DS_2_-VASc scores with LAT in patients with NVAF. Because the predictive power of the CHADS_2_ and CHA_2_DS_2_-VASc scores for the presence of LAT in NVAF is modest (*c*-statistics 0.55~0.70) [9–13], we further sought to develop a new scoring system that might improve prediction of the presence of LAT as a surrogate for cardioembolic risk in NVAF patients.

## 2. Methods

### 2.1. Study Patients

We retrospectively evaluated 2,826 consecutive atrial fibrillation patients who underwent a TEE at the Guangdong General Hospital to screen for LAT before ablation or cardioversion. Of these, 131 patients were excluded because of histories of rheumatic heart disease or prosthetic valve placement. The remaining 2695 patients were considered to have NVAF and were included in our analysis. Ethical approval was obtained from the Guangdong General Hospital Medical Center Institutional Review Board.

### 2.2. Assessment of CHADS_2_ and CHA_2_DS_2_-VASc Score

CHADS_2_ score was determined by assigning 1 point each for the presence of congestive heart failure (CHF), hypertension, age ≥ 75 years, and diabetes and by assigning 2 points for previous stroke/transient ischemic attack (TIA) [14]. CHA_2_DS_2_-VASc score was determined by assigning 1 point each for the presence of CHF, hypertension, age 65 to 74 years, diabetes, and vascular disease (peripheral artery disease or myocardial infarction) and by assigning 2 points each for age ≥ 75 years and previous stroke/TIA [15]. CHF was defined as the existence of clinical manifestations of heart failure within the last 3 months, with or without left ventricular systolic dysfunction, as previously described [16]. According to the CHADS_2_ or CHA_2_DS_2_-VASc risk scores, patients were classified as low (CHADS_2_ or CHA_2_DS_2_-VASc score = 0), intermediate (CHADS_2_ or CHA_2_DS_2_-VASc score = 1), or high (CHADS_2_ or CHA_2_DS_2_-VASc score ≥ 2) risk groups. Patients were instructed to stop taking warfarin at the time of admission to the hospital and then treated with subcutaneous low molecular weight heparin until 12 hours before ablation.

### 2.3. Echocardiography

Transthoracic and transesophageal echocardiography were performed using commercially available equipment (Vivid 7 or E9, GE Medical Systems, Milwaukee, WI). Transthoracic echocardiography was performed with a 2.5 or 3.5 MHz phased-array transducer. TEE was performed with a 5 MHz multiplane transducer. Each patient was examined after overnight fast and without premedication except for topical anesthesia of the hypopharynx with lidocaine spray. For TEE examination, multiple standard tomographic planes were imaged. Thrombus was defined as a circumscribed, uniformly echo dense mass distinct from the underlying left atrial endocardium and pectinate muscles [17]. Spontaneous echocardiographic contrast (SEC) was defined as dynamic “smoke-like” echoes with characteristic swirling motion that could not be eliminated despite optimized gain settings [18]. We defined moderate to severe left ventricular systolic dysfunction as having an LVEF ≤ 40% according to 2010 European Society of Cardiology guidelines [3]. Left atrial size was categorized into 2 groups according to left atrial diameter and sex: normal left atrial size in mm (women, ≤38; men, ≤40) and left atrial enlargement (LAE) in mm (women, >38; men, >40) [19].

### 2.4. Statistical Analysis

Continuous variables were presented as mean and standard deviation (SD) and categorical variables as frequencies and percentages. Chi-square test was used to compare the presence of LAT by groups of CHADS_2_/CHA_2_DS_2_-VASc scores. A two-tailed *P* < 0.05 was considered statistically significant. Multivariate logistic regression was used to examine the association of clinical and transthoracic echocardiographic parameters with the presence of LAT. We also assessed the discriminative ability of the CHADS_2_ and CHA_2_DS_2_-VASc by receiver operating characteristic (ROC) analysis giving area under curve (AUC) summary statistic (*c*-statistic). All statistical analyses were performed using SPSS for Windows (Version 19.0, SPSS Inc., Chicago, IL, USA).

## 3. Results

Table 1 shows that of 2,695 patients, the mean (SD) age was 57.8 (11.8) years. Most were men (67.7%), 16.2% had nonparoxysmal AF, 27.7% had LAE, 1.2% had moderate to severe left ventricular systolic dysfunction, and 2.6% had cardiomyopathy (72.5% were hypertrophic cardiomyopathy; 17.4% were dilated cardiomyopathy). Most of patients were in a low-risk group defined as CHADS_2_ (45.8% and 36.0% had score of 0 and 1, respectively) or CHA_2_DS_2_-VASC scores less than 2 (27.0% and 31.7% had score of 0 and 1, respectively).

Table 2 shows that of 2,695 patients, 81 (3.0%) had LAT. LAT was found in 2.2% patients with CHADS_2_ score of 0, 3.6% with CHADS_2_ score of 1, and 3.9% with CHADS_2_ score of 2 or more. Results regarding the CHA_2_DS_2_-VASc score showed that the prevalence of LAT was 1.9% in those with score of 0, 3.2% in those with score of 1, and 3.6% in those with score of 2 or more. Neither the CHADS_2_  (*P* = 0.07) nor the CHA_2_DS_2_-VASc score (*P* = 0.12) was significantly associated with the presence of LAT.

Table 3 shows that, among components of the CHADS_2_/CHA_2_DS_2_-VASc score, hypertension (OR 1.65, 95% CI 1.03–2.65) and previous stroke/TIA (OR 3.13, 95% CI 1.49–6.57) were significant predictors for LAT (Model 1). Additionally adjusting for other conventional risk factors such as nonparoxysmal AF, moderate to severe left ventricular systolic dysfunction, LAE, coronary heart disease, and cardiomyopathy in the model showed that, of components in the CHADS_2_/CHA_2_DS_2_-VASc score, only previous stroke/TIA (OR 2.90, 95% CI 1.31–6.40) was significantly associated with the presence of LAT. Other significant predictors included nonparoxysmal AF (OR 1.83, 95% CI 1.10–3.03), moderate to severe left ventricular systolic dysfunction (OR 3.51, 95% CI 1.07–11.5), LAE (OR 3.77, 95% CI 2.28–6.25), and cardiomyopathy (OR 3.18, 95% CI 1.42–7.05) (model 2 of the Table 3).

ROC analysis showed that the AUC concerning LAT prediction using CHADS_2_ and CHA_2_DS_2_–VASc was 0.574 (95% CI 0.514–0.634, *P* < 0.001) and 0.569 (95% CI 0.507–0.631, *P* = 0.001), respectively. No significant difference in discrimination between the CHADS_2_ and CHA_2_DS_2_-VASc for LAT was found (*P* = 0.90). A new composition model including the risk factors that were significantly associated with the presence of LAT (previous stroke/TIA, nonparoxysmal AF, moderate to severe left ventricular systolic dysfunction, LAE, and cardiomyopathy) improved the discrimination significantly (AUC = 0.743, 95% CI 0.689–0.798). *P* value for comparing the difference between the new composition model and the CHADS_2_/CHA_2_DS_2_-VASc was <0.001.

## 4. Discussion

Our study showed that the ability of both CHADS_2_ and CHA_2_DS_2_-VASc scores in predicting LAT was consistently limited. As NVAF patients might be of higher risks for LAT despite a low CHADS_2_/CHA_2_DS_2_-VASc score, we therefore developed a new composition model, which included parameters of previous stroke/TIA, nonparoxysmal AF, moderate to severe left ventricular systolic dysfunction, LAE, and cardiomyopathy and showed that the new composition score significantly increased the discrimination (*c*-statistics from 0.57 to 0.74), suggesting that combining the clinical and echocardiographic parameters might be of important clinical significance in terms of predicting LAT, which has been well used as a surrogate for cardioembolic risk in NVAF patients.

Stroke in patients with atrial fibrillation is usually considered as thromboembolism due to LAT. Almost all guidelines focus on anticoagulation in order to reduce the thromboembolism risk of NVAF. CHADS_2_ and CHA_2_DS_2_-VASc scores have been widely used to predict the risk of stroke in patients with atrial fibrillation. However, the relationship between CHADS_2_/CHA2DS_2_-VASc score and LAT was still unclear. In our study, neither the CHADS_2_ nor the CHA_2_DS_2_-VASc score was significantly associated with the presence of LAT, suggesting a limited predictive value for LAT. Our findings were not inconsistent with the majority of previous studies showing a modest predictive value for LAT by using CHADS_2_/CHA_2_DS_2_-VASc score (*c*-statistics 0.55~0.7) [9–13]. Several studies suggested that the CHADS_2_/CHA_2_DS_2_-VASc score was not independent risk factors for LAT [1, 20, 21], although some reported a positive association between the CHADS_2_/CHA_2_DS_2_-VASc score and the risk of LAT [2, 20, 22–28]. The exact explanations for the variation in the results of these studies were unclear. Possible explanations include the variation in AF duration, coexistent structural cardiac abnormalities, race, adequacy of anticoagulation, and other common cardiovascular risk factors. Our study suggested that the predictive ability of CHADS_2_/CHA_2_DS_2_-VASc score on stroke was unlikely mainly through the mechanism of cardiogenic embolism. Theoretically, the same composition score may be comprised of different components, and even for the same composition score, because of the variability of disease duration and the severity of its individual components, the effects on thrombus formation might be different. The CHADS_2_ and CHA_2_DS_2_-VASc scores were thus logically unlikely to have an accurate prediction.

As most components of the CHADS_2_ and CHA_2_DS_2_-VASc scores are risk factors for atherosclerosis, the atherothrombotic pathway may partly explain the positive association of CHADS_2_/CHA_2_DS_2_-VASc score with stroke in patients with NVAF [6–8]. A recent study showed that the CHADS_2_ and CHA_2_DS_2_-VASc scores were not associated with the stroke phenotype according to diffusion-weighted imaging lesion volumes and patterns in AF patients [29]. Notably, considering patients in non-AF populations, such as acute coronary syndrome [30], sick sinus syndrome [31], and community population [32, 33], the discriminatory performance of the CHADS_2_/CHA_2_DS_2_-VASc scores in predicting ischemic stroke/TIA events was similar or even better in patients without, rather than with, AF, which further indicated that CHADS_2_/CHA_2_DS_2_-VASc scores may predict stroke in NVAF patients through the mechanism of noncardiogenic embolism and most likely the mechanism of atherosclerosis. This has an important clinical implication. For NVAF patients with high CHADS_2_/CHA_2_DS_2_-VASc score, we might need to not only target anticoagulation, but also emphasize good management of atherosclerotic risk such as blood pressure, diabetes, cholesterol, and other risk factors, which was often overlooked in stroke prevention in NVAF patients. Perindopril Protection Against Recurrent Stroke Study (PROGRESS) has clearly demonstrated that blood pressure-lowering therapy reduces the risk of major vascular events in patients with atrial fibrillation and prior stroke or TIA [34].

As shown in our study and previous studies with large sample size [21, 26], NVAF patients may have a risk of LAT despite a low CHADS_2_/CHA_2_DS_2_-VASc score. It is unreasonable to consider TEE not necessary in NVAF patients with a CHADS_2_/CHA_2_DS_2_-VASc score of 0 before ablation or cardioversion. According to 2014 AHA/ACC/HRS Guideline for the Management of Patients with Atrial Fibrillation [4], NVAF patients with CHA_2_DS_2_-VASc score less than 2 were categorized as having low-intermediate-risk for stroke and thus were not recommended to receive oral anticoagulant therapy. These patients may be exposed to the high risk of stroke of about 10% due to LAT [35]. Risk stratification schemes based on the risk factors of LAT would help to identify such patients. On the other hand, antithrombotic strategies were not consistently used between patients with cardiogenic embolism and noncardiogenic embolism. Establishing specific stratification schemes predictive of cardioembolic stroke risk may enable assignment of NVAF patients to the most beneficial anticoagulant therapy. However, presently we could not reliably distinguish cardioembolic stroke from noncardioembolic ischemic stroke on the basis of clinical and imaging features in AF patients [36]. Therefore it is unlikely to use prospective cohort studies to explore new risk stratification schemes with cardioembolic stroke as a clinical endpoint. LAT is the material basis of cardiogenic embolism in atrial fibrillation. It may be a useful way to establish potential risk stratification models specifically for cardioembolic stroke due to LAT, based on the risk factors of LAT. In our study, previous stroke/TIA, nonparoxysmal AF, moderate to severe left ventricular systolic dysfunction, LAE, and cardiomyopathy were independent risk factors for LAT. A new composition model including them significantly increased the discrimination for LAT compared with CHADS_2_/CHA_2_DS_2_-VASc score. In fact, nonparoxysmal AF [37], left ventricular systolic dysfunction [38], and LAE [38, 39] have been shown to be independent risk factors for stroke in NVAF in other studies. The predictive value of our new composition score for cardiogenic embolism due to LAT in NVAF needs further validation.

There were several limitations in our study. First, this is a single center retrospective study and may not reflect the experience of other settings. Second, there was heterogeneity of aggressiveness of anticoagulation and/or target-achieved INR before ablation or cardioversion, which might be closely related to thrombosis. The duration of atrial fibrillation, heart rhythm of patient with thrombus detected, biomarkers such as brain natriuretic peptide, serum creatinine, troponin and uric acid, and parameters of left atrial appendage emptying fraction or flow velocity which may affect the presence of LAT were not included in the analysis because some of the information was missing in our patients.. In addition, data on left atrial volume, a more accurate marker to assess left atrial size, was also not available in the current study. Third, classification of heart failure based on the New York Heart Association classification may be subjective. Finally, our subjects were patients who had TEE performed in preparation for treatment of NVAF by ablation or cardioversion. Most patients included have a low score (0–2). Therefore, our results may not be fully representative of all patients with NVAF.

In conclusion, based on a large hospital-based sample, we showed a limited predictive ability of CHADS_2_/CHA_2_DS_2_-VASc score for LAT in NVAF patients, suggesting the CHADS_2_/CHA_2_DS_2_-VASc score might not predict stroke mainly through the cardiogenic embolism pathway. Combining clinical and echocardiographic parameters significantly improved the discriminatory ability for LAT. However, the predictive value of this new composition model for cardiogenic embolism due to LAT in NVAF patients needs further validation.

## Figures and Tables

**Table 1:** Characteristics of 2,695 patients who performed transesophageal echocardiogram before ablation and cardioversion.

Characteristic	Mean ± SD or number (%)
Age, years	57.8 ± 11.8
Age groups	
<65	1848 (68.6)
65–74	708 (26.3)
≥75	139 (5.2)
Men	1824 (67.7)
Nonparoxysmal AF	437 (16.2)
Congestive heart failure	446 (16.5)
Hypertension	1075 (39.9)
Diabetes mellitus	273 (10.1)
Stroke/TIA/systemic embolic event	98 (3.6)
Vascular disease	39 (1.4)
Cardiomyopathy	69 (2.6)
Coronary heart disease	196 (7.3)
CHADS_2_	
0	1234 (45.8)
1	971 (36.0)
2	344 (12.8)
3	114 (4.2)
4	27 (1.0)
5	5 (0.2)
CHA_2_DS_2_-VASC	
0	728 (27.0)
1	855 (31.7)
2	592 (22.0)
3	317 (11.8)
4	135 (5.0)
5	50 (1.9)
6	16 (0.6)
7	2 (0.1)
PT-INR at the time of TEE	1.22 ± 0.47
<1.5	2278 (84.5%)
1.5–2.0	215 (8.0%)
>2.0	202 (7.5%)
Left atrial spontaneous echo contrast	124 (4.6%)
LAT	81 (3.0%)
LAD (mm)	36.9 ± 5.9
LAE	747 (27.7%)
LVEF%	65.1 ± 7.4
LVEF% ≤ 40%	32 (1.2%)

AF = atrial fibrillation; TIA = transient ischemic attack; CHADS2 = congestive heart failure, hypertension, age ≥ 75 years, diabetes mellitus, and previous stroke/transient ischemic attack [double risk weight]; CHA2DS2-VASc = congestive heart failure, hypertension, age ≥ 75 years [doubled risk weight], diabetes mellitus, previous stroke/transient ischemic attack [doubled risk weight], vascular disease, age 65 to 74 years, and sex; TEE = transesophageal echocardiogram; LAT = left atrial thrombus; LAE = left atrial enlargement; LVEF = left ventricular ejection fraction.

**Table 2:** Presence of LAT by CHADS_2_ or CHA_2_DS_2_-VAS scores.

Risk category	Total number	LAT, *n* (%)	*P* values (*χ*^2^ test)
*CHADS*_*2*_ * score*			
0	1234	27 (2.2)	0.07
1	971	35 (3.6)	
2+	490	19 (3.9)	
*CHA*_*2*_ *DS*_*2*_ *-VASc score*			
0	728	14 (1.9)	0.12
1	855	27 (3.2)	
2+	1112	40 (3.6)	

LAT = left atrial thrombus; CHADS_2_ = congestive heart failure, hypertension, age ≥ 75 years, diabetes mellitus, and previous stroke/transient ischemic attack [double risk weight]; CHA_2_DS_2_-VASc = congestive heart failure, hypertension, age ≥ 75 years [doubled risk weight], diabetes mellitus, previous stroke/transient ischemic attack [doubled risk weight], vascular disease, age 65 to 74 years, and sex; TEE = transesophageal echocardiogram.

**Table 3:** Adjusted odds ratios (ORs) and 95% confidence interval (CI) of left atrial (LA) thrombus for specific risk factors in the CHADS_2_ and CHA_2_DS_2_-VASc scores.

Risk factors	Model 1	Model 2
Congestive heart failure	0.85 (0.45–1.59)	0.58 (0.29–1.18)
Hypertension	1.65 (1.03–2.65)^*∗*^	1.42 (0.86–2.35)
Age, years		
<65	Ref.	
65–74	1.36 (0.41–4.51)	1.41 (0.42–1.53)
≥75	2.30 (0.69–7.64)	2.29 (0.67–7.78)
Diabetes	0.84 (0.41–1.74)	0.73 (0.34–1.56)
Previous stroke/TIA	3.13 (1.49–6.57)^*∗∗*^	2.90 (1.31–6.40)^*∗*^
Vascular disease	0.73 (0.10–5.46)	0.89 (0.12–6.87)
Female	0.72 (0.44–1.19)	0.60 (0.35–1.01)
Nonparoxysmal AF	—	1.83 (1.10–3.03)^*∗*^
LVEF% ≤ 40%	—	3.51 (1.07–11.5)^*∗*^
LAE	—	3.77 (2.28–6.25)^*∗∗∗*^
Coronary heart disease	—	1.59 (0.75–3.38)
Cardiomyopathy	—	3.18 (1.42–7.05)^*∗∗*^

TIA = transient ischemic attack; AF = atrial fibrillation; LVEF = left ventricular ejection fraction; LAE = left atrial enlargement

^*∗*^ *P* < 0.05; ^*∗∗*^*P* < 0.01; ^*∗∗∗*^*P* < 0.001.
